# Clearing the Air: Understanding the Impact of Wildfire Smoke on Asthma and COPD

**DOI:** 10.3390/healthcare12030307

**Published:** 2024-01-25

**Authors:** May-Lin Wilgus, Maryum Merchant

**Affiliations:** Department of Medicine, David Geffen School of Medicine, University of California Los Angeles, Los Angeles, CA 90095-1405, USA; mmerchant@mednet.ucla.edu

**Keywords:** wildfire, smoke, particulate matter, pollution, respiratory, asthma, chronic obstructive pulmonary disease (COPD), climate change, planetary health

## Abstract

Wildfires are a global natural phenomenon. In North America, wildfires have not only become more frequent, but also more severe and longer in duration, a trend ascribed to climate change combined with large fuel stores left from modern fire suppression. The intensification of wildfire activity has significant implications for planetary health and public health, as exposure to fine particulate matter (PM_2.5_) in wildfire smoke is linked to adverse health effects. This review focuses on respiratory morbidity from wildfire smoke exposure. Inhalation of wildfire PM_2.5_ causes lung injury via oxidative stress, local and systemic inflammation, airway epithelium compromise, and increased vulnerability to infection. Wildfire PM_2.5_ exposure results in exacerbations of pre-existing asthma and chronic obstructive pulmonary disease, with an escalation in healthcare utilization, including emergency department visits and hospitalizations. Wildfire smoke exposure may be associated with asthma onset, long-term impairment of lung function, and increased all-cause mortality. Children, older adults, occupationally-exposed groups, and possibly women are the most at risk from wildfire smoke. Future research is needed to clarify best practices for risk mitigation and wildfire management.

## 1. Introduction

Wildfires are a natural part of the ecological cycle termed the “fire regime” [1]. The last few decades have witnessed a concerning surge in the frequency, size, and duration of wildfires [2]. Despite efforts to suppress wildfires, ecological and human-induced factors contribute to the intensification of wildfires. Wildland vegetation accumulates until fire is unavoidable unless managed through thinning, grazing, or prescribed burns [3]. Landscapes are drier due to rising global temperatures and prolonged drought periods, creating greater fuel for wildfires. Changes in atmospheric circulation patterns and winds further contribute by influencing the speed and spread of wildfires. Human activities such as fire suppression, expansion of the wildland-urban interface, and accidental ignitions have created conditions that make wildfires more severe when they do occur. The Euro-American practice of fire exclusion, intended to reduce low-intensity fires, paradoxically resulted in the accumulation of large fuel stores, leading to more intense and catastrophic fires [4]. It is noteworthy that from 1979 to 2022, the mean duration of the global fire season increased by 18.7%, stressing the evolving nature of this problem [5]. In 2021, wildfires reached an alarming scale, resulting in a loss of 9.3 million hectares of tree cover globally. 2023 saw heightened fire activity, including record-breaking burns across Canada and catastrophic fires in Hawaii [6]. A recent report by the United Nations Environment Programme (UNEP) highlights the escalating threat, projecting a global increase of extreme fires by 14% by 2030, 30% by the end of 2050, and 50% by the end of the century. The report also found an elevated risk for large fires even in previously unaffected areas, including the Arctic [7].

Wildfires emit particulate matter and other air pollutants and are the source of an estimated 26% of summertime organic aerosols in the western United States [8]. Although there was an overall reduction in pollution levels in the United States following the implementation of the Clean Air Act, the prevalence of wildfire pollution is on the rise [1]. This has enormous economic consequences: the health costs of wildfire smoke are estimated between $11–20 billion annually in the US alone [9]. Globally, wildfire smoke is estimated to cause over 339,000 premature deaths a year—far more than those who lose their lives directly in these blazes [10]. The trajectory of rising wildfire incidence and severity is expected to continue, and it is predicted by 2050 the fire season will be 23 days longer than the 2013 season [11]. Given the trend in wildfires along with a decrease in ambient pollution emissions, over 50% of particulate pollution will likely be caused by fires by the end of the century [12].

The rise in wildfire activity has significant implications for public health, as exposure to wildfire smoke is linked to adverse health effects [13]. The evidence for the effects of wildfire smoke on respiratory disease is the most compelling and is the focus of this review. To identify articles on the topic, PubMed was interrogated up to January 2024 using the search terms “wildfire”, “smoke”, “air pollution”, “particulate matter”, “PM_2.5_”, “asthma”, “COPD”, “chronic obstructive pulmonary disease”, “small airways”, and “lung cancer”. Furthermore, the reference lists of review papers and meta-analyses were searched for additional relevant articles.

## 2. Toxic Exposures Created by Wildfires

Air pollution is divided into indoor (household) pollution and outdoor (ambient) pollution; during a wildfire, the smoke contributes to total ambient air pollution. The vegetation in wildlands, composed of trees, bushes, grasses, and peat, acts as the fuel for wildfire smoke [1]. Fires at the wildland-urban interface (WUI), an evolving region that has increased by 62% from 2000 to 2016 [14], burn structures and vehicles in addition to biomass; these urban fuels can add even more toxic species to the emitted aerosols [15]. The pollutants generated by wildfires include fine and coarse particulate matter (PM), carbon monoxide, methane, nitrous oxides, and volatile organic carbons (VOC), as well as ozone (O_3_) from the reaction of nitrogen oxides and VOCs in the atmosphere [1,16]. Wildfire smoke also contains at least 20 types of HAPs recognized by the US Environmental Protection agency, such as formaldehyde, benzene, and hydrogen cyanide [17,18]. Although carbon monoxide exposure is usually confined to areas directly affected by a fire, the particulate matter spreads much further [19]. The particulate matter from wildfires can be more toxic than ambient non-smoke pollution particulates due to greater exposure peaks, more intensive cumulative exposure, and differences in composition [20]. Wildfires generate fine (under 2.5 microns) and ultrafine (under 1 micron) particulates, commonly known as PM_2.5_, which disperse over a greater area and settle more slowly than larger particles, resulting in prolonged exposure time and sizable affected regions [21]. Wildfire PM_2.5_ can impact residents in a 10-to-15-times-larger area than the actual area burned [5]. Fine and ultrafine particulates are unique in their ability to not only penetrate deeply into the lungs causing local damage, but can also be absorbed into the bloodstream causing systemic harm [22]. Wildfire smoke contains more oxidative components, such as polycyclic aromatic hydrocarbons and quinones, along with pro-inflammatory elements, such as aldehydes and oxides of nitrogen, rendering it more toxic than the non-smoke pollution particulates in urban areas [20]. Moreover, the high temperatures, characteristic of wildfires, facilitate the oxidation of the smoke particles, giving rise to free radicals. This amplifies the risk of cellular damage inflicted on cells and tissues within the human body [23,24].

The US National Ambient Air Quality recommends a 24-h PM_2.5_ exposure of less than 35 µg/m^3^, and the World Health Organization recommends daily PM_2.5_ exposure not exceed 25 µg/m^3^; however, in wildfire-affected areas, the PM_2.5_ concentration can far exceed these guidelines. A study conducted in Alberta, Canada, revealed that during wildfire, the PM_2.5_ may increase to over 50 µg/m^3^, in contrast to their typical baseline of 3–6 µg/m^3^ [25]. An analysis of hospital visits during California wildfires demonstrated a median PM_2.5_ exposure difference of 23.4 µg/m^3^ between fire and non-fire days, with some days exceeding 80 µg/m^3^ [26]. The intensity of exposure can be even greater: a 24-h concentration of nearly 400 µg/m^3^ from wildfire smoke has been documented [27]. Between 2008 and 2012, about 10.3 million individuals in the United States experienced unhealthy air quality levels (average daily PM_2.5_ exceeding 35 µg/m^3^) for more than 10 days due to exposure to wildfires [28]. 

## 3. Mechanisms of Lung Injury from Wildfire Smoke Exposure

Small airways, which are airways <2 mm in diameter without cartilage and include respiratory bronchioles, are an important site of pathology in asthma and COPD [29]. While the small airways contribute little to airway resistance in healthy individuals [30], in people with obstructive airway disease small airways resistance can increase by 40-fold [29]. Small airway obstruction occurs via increased submucosal thickness from inflammation, fibrosis of airway walls, increase in smooth muscle mass, mucus in the airway lumen, and distortion from loss of alveolar attachments [31]. The lower airways are especially susceptible to injury from inhaled particulate matter: the smaller diameter of peripheral airways means that particulates can more easily contact the surface and cause damage [29]. PM_2.5_ likely drives low-grade inflammation in the small airways, initiating bronchiolitis [32]. In contrast to tobacco smoke, biomass smoke exposure is exclusively associated with small airway inflammation rather than emphysema [33]. Proposed mechanisms for wildfire smoke injury to the lungs include disruption of the epithelial barrier, oxidative stress, an altered response to infection, epigenetic changes, shift of the Th1/Th2 immune response, and coincident increases in allergen exposures with wildfires [34]. Systemic effects of wildfire smoke exposure may also occur via several potential mechanisms: particulate matter interacting with neural receptors in the lungs and activating the autonomic nervous system; a local and systemic immune response to pollutants in the alveoli; and direct translocation of ultrafine particulate matter across the alveolar membrane resulting in systemic endothelial dysfunction, activation, and injury [1].

Air pollutants such as wildfire smoke have notable effects on airway epithelium and obstructive lung disease. The airway epithelium normally serves as a physical barrier and an immunologic barrier via mucociliary clearance and production of immune mediators. Damage to the airway epithelial barrier by inhaled pollutants disrupts epithelial cell-cell contact, a mechanism of airway injury that is linked with obstructive lung disease [22]. Airway epithelial disruption also leads to the release of immune mediators, and an exaggerated inflammatory response has been correlated with airway remodeling and disease pathogenesis and progression in asthma and COPD [22]. Studies have shown an association between PM_2.5_ exposure and the initiation of epithelial-mesenchymal transition (EMT), the process by which epithelial cells lose apical-basal polarity and acquire a mesenchymal phenotype, thereby allowing for migration, invasion, and production of extracellular matrix [35]. EMT is associated with tissue fibrosis and carcinogenesis. Airway remodeling after PM_2.5_-mediated lung injury is controlled in part by myofibroblasts derived from epithelial cells that undergo EMT, and the process can lead to scarring and changes in tissue architecture, as well as goblet cell metaplasia, basal cell hyperplasia, and squamous metaplasia [29]. PM_2.5_ also reduces mucociliary clearance; in vitro studies show airway epithelial cells exposed to high concentrations of PM_2.5_ have reduced ciliary beat, attenuated cilia, and increased mucus marker gene expression [36,37]. Mucus hypersecretion blocks airways and can harbor microbes that further induce tissue inflammation [29]. 

A compromised airway epithelium enhances pathogen entrance, increasing the risk for infection, which is an important pathway to exacerbation and, thus, progression of obstructive airway disease [22]. Wildfire PM_2.5_ exposure compromises the immune response and appears to increase the morbidity and mortality from infectious disease for weeks after the exposure [38,39]. Exposure to PM_2.5_ in experimental animals leads to increased diversity of the lung microbiome [40], and greater lung microbiome diversity has been associated with respiratory diseases, including asthma [41]. Some evidence suggests PM_2.5_ can even carry airborne microbes into the lungs [42]. The bioaerosols created by wildfires can transmit fungal and bacterial cells and affect allergic and inflammatory lung disease [43]. The incidence of some infections, such as coccidioidomycosis and aspergillosis, increases after wildfire events [44]. The water vapor, shelter from ultraviolet radiation, and carbon material in smoke all serve to potentially preserve microbes during wildfire-associated transport. Microbial cell counts in smoke from prescribed fires are five-times that of background levels [43]. 

Experimental data support the theory that wildfire smoke leads to lung injury via inflammation and oxidative stress [45]. In vitro studies demonstrate that wildfire-derived PM_2.5_ increases inflammatory gene expression in human bronchial epithelial cells and is cytotoxic to macrophage cell lines [45,46]. In vivo data show wildfire-derived PM exposure leads to oxidative stress-mediated macrophage toxicity and that human alveolar macrophages release pro-inflammatory cytokines (TNF-α, IL-6, IL-1ß, and GM-CSF) in response to incubation with components of smoke-derived pollution [47,48]. In a mouse model, exposure to biomass particles results in significant lung injury. Biomass pollution causes an inflammatory influx into the lung parenchyma and results in a worse impact on airway mechanics than traffic-derived pollution [49]. Human studies confirm these findings. Human exposure to wildfire pollutants is associated with a significant increase in peripheral band neutrophils and an elevation in cytokine levels, suggesting a systemic immune response [48,50]. In a controlled human study examining exposure to woodsmoke PM_2.5_ during treadmill exercise, markers of inflammation and oxidative stress in exhaled breath condensate and plasma were elevated post-exposure [51]. An increase in inflammatory markers (IL-6, IL-8) was also observed in volunteer firefighters after a 12-h wildfire suppression shift [52]. Wildfire health effects may also occur via epigenetic change. In a twin study, exposure to wildfire PM_2.5_ was associated with long-term global DNA methylation changes distinct from changes associated with non-smoke-related PM_2.5_ [53]. Taken together, the data support inflammation, oxidative stress, airway epithelial injury with epithelial-mesenchymal transition, and epigenetic change as biologically plausible mechanisms for respiratory illness from exposure to PM_2.5_ emitted by wildfires.

In addition to lung injury, ambient air pollution has also been associated with carcinogenesis and risk for lung cancer. Pollutants such as PAHs cause DNA adducts and epigenetic modification [54]. In vitro, combustion-derived particles from wood-burning alter cell proliferation and cause DNA damage, suggesting these particles are involved in early cancer development [55]. Chronic exposure to PM_2.5_ enhances invasion in lung adenocarcinoma cell lines and promotes tumorigenesis and metastasis in patient-derived xenograft models [56]. It was recently demonstrated that PM_2.5_ promotes lung cancer by acting on cells in healthy tissue that harbor pre-existing oncogenic mutations such as EGFR mutations [57]. There is little oncologic research specific to wildfire smoke exposures, so further study is needed to evaluate the distinct carcinogenic effects of wildfire smoke. 

## 4. Epidemiological Evidence for the Effects of Wildfire Smoke on Airways Disease

Evidence-based data regarding the adverse health effects of wildfire smoke is limited by the lack of control populations or long-term studies. In addition, some epidemiologic research combines respiratory diagnoses (for example, asthma, COPD, and bronchitis) and outcomes (such as emergency department visits and hospitalizations) for analysis, which may limit interpretation. Research into the health effects of wildfire smoke is also influenced by the methods used to estimate exposure [1], and there is no universally accepted standard for approximating exposure on a population level. Various approaches are used to estimate wildfire smoke exposure, including area monitor PM measurements, modeled PM, satellite data, chemical transport models, and a combination of these techniques [58]. Temporal comparisons are confounded by temperature and humidity, and area monitors can be imprecise due to spatial variation in smoke [8]. The diversity in methodologies highlights the complexity of studying the health impacts of wildfire smoke. Nevertheless, there is substantial compelling epidemiologic data correlating adverse respiratory outcomes with wildfire smoke exposure [Table 1]. Several studies confirm the respiratory risks associated with wildfire smoke-related PM_2.5_ are distinct from the risks of non-smoke ambient PM_2.5_ exposure and from co-pollutants such as ozone [26,59,60].

Wildfire smoke exposure is strongly associated with asthma symptoms, healthcare utilization, emergency department visits, and hospitalizations. The majority of studies looking at wildfire smoke exposure and asthma emergency department visits have shown a positive association [13,25,26,59,61,62,66,67,68,69], although Tinling et al. showed no significant association [83]. Likewise, the bulk of data regarding asthma hospitalizations after wildfire exposure demonstrates a significant increase in hospitalizations [13,59,61,62,63,64]. A 10 µg/m^3^ increase in PM_2.5_ is associated with an 11% increase in asthma-related emergency department visits [68]. Reid et al. found a linear increase in asthma hospitalizations for wildfire exposure, with a relative risk of 1.07 (95% CI 1.05–1.1) for every 5 µg/m^3^ increase in wildfire PM_2.5_ exposure [13]. Malig et al. demonstrate that fire-related PM_2.5_ is more tightly linked with asthma emergency department visits (RR 1.46, 95% CI 1.38–1.55) than non-fire PM_2.5_ (RR 0.77, 95% CI 0.55–1.08) [26]. Heaney et al. show that “smoke event days” (defined as >98th percentile daily wildfire PM_2.5_ exposure) are associated with a 3.3% increase in hospital visits for all respiratory conditions and a 10.3% increase in asthma hospital visits [25]. Doubleday’s group in Washington found a significant correlation between wildfire and emergency department visits for asthma as well as all respiratory conditions. This increase in healthcare utilization persisted for five or more days after the initial exposure to smoke [69]. A systematic review confirmed a significant increase in all respiratory emergency department visits and asthma-related hospitalizations within the first 3 days of exposure to wildfire smoke [70]. Taken together, the aggregated relative risk for elevated short-term effects of wildfire smoke on asthma is around 1.1 (range 1.07–1.68 in reported studies) per 10 µg/m^3^ rise in wildfire PM_2.5_ [34]. 

Aside from exacerbations requiring hospitalization, most evidence also shows an adverse effect of smoke exposure on less severe asthma symptoms. There is an association between increased asthma reliever medication use and oral corticosteroid prescriptions [71]. Mahsin et al. show a significant uptick in physician visits for asthma after wildfire exposure [72]. Within an Australian severe asthma registry, wildfire smoke exposure was associated with respiratory symptoms, including breathlessness, wheezing, and cough. Sixty-five percent of the registrants described an inability to particulate in usual activities, and seventy-three percent increased their use of asthma reliever inhalers. Although most followed instructions for staying indoors and closing windows, this did not mitigate the symptoms. In addition, the worsening of symptoms and quality of life persisted for months after the exposure. In this study the use of mepolizumab was protective against asthma exacerbations during wildfire events [73]. However, there exists conflicting data regarding wildfire smoke and asthma symptoms, as Lipner’s group at National Jewish Health found no association between wildfire smoke exposure and asthma control test scores in a pediatric asthma clinic [60]. 

The evidence linking wildfire-specific smoke exposure and COPD exacerbations is less strong than the asthma evidence, but overall consensus suggests a significant association [8]. Some studies have shown significant associations between wildfire smoke exposure and COPD emergency department visits [13,26,75] or hospitalizations [13,61,63,64], although there have been several negative studies [62,66,83]. An analysis of California wildfires showed an increase in emergency department visits for COPD during fire periods, with a relative risk of 1.18 [26]. Gan et al.’s data show that the measured effect on COPD hospitalization varies by the means of estimating smoke PM_2.5_ exposure [61]. Magzamen et al. confirm a 10 µg/m^3^ increase in wildfire PM_2.5_ is associated with greater COPD hospitalizations 3 days after the exposure [64]. Symptom flares post-wildfire exposure are described in the COPD population: in a prospective study of COPD patients in the Denver region, days characterized by heightened exposure to wildfire smoke were associated with worse COPD symptom scores. Notably, these scores returned to baseline levels after the exposure [76]. 

There is evidence that the respiratory consequences of wildfire exposure are not limited to acute effects. Wildfire exposure can lead to long-term effects on asthma: an observational study using clinical data from an outpatient allergy clinic detected persistent decreases in respiratory peak flow a year after the wildfire event. The group used complex meteorological data to discern that with each 1% escalation in wind directing the fire toward the community, there was an associated decrease of 2.21 L/min in peak flow. Potential theories for the long-term effects of wildfire smoke on asthma include increasing allergic sensitization to allergens and altering immune responses [77]. A study in Indonesia found a significant reduction in lung function in adults, but not children, 10 years after exposure to a major wildfire [78]. However, since lung development continues into the postnatal period, children are theoretically susceptible to the effects of wildfire smoke exposure on lung function. Wildfire PM_2.5_ emission exposures were linked to declines in lung function in non-asthmatic children, with some authors theorizing that asthma medication use in asthmatic children prevented loss of lung function [8,84]. Research involving infant rhesus monkeys exposed to ambient wildfire smoke demonstrated long-term impairment in lung function after early-life wildfire smoke exposure [85]. Wildfire exposure may also be a risk factor for asthma development in previously healthy individuals. New onset asthma post-wildfire smoke exposure was described in firefighters in Alberta, Canada, where higher estimated exposure to fire was associated with a greater incidence of bronchial wall thickening and bronchoreactivity by methacholine challenge testing [74].

The 2023 Global Initiative for Chronic Obstructive Lung Disease (GOLD) statement on COPD and air pollution describes ambient and indoor air pollution as a risk factor for COPD development, accelerated decline in lung function in people with COPD, and impairment of lung development in children [86]. As a substantial and particularly toxic component of ambient air pollution, it can be inferred that wildfire-specific PM_2.5_ is a risk factor for impaired lung function; however, further studies are needed to determine the long-term effects of acute and/or repeated exposure to wildfire smoke in developing lungs, healthy adults, and individuals with chronic lung disease.

## 5. Other Adverse Health Effects of Wildfire Smoke Exposure

There is growing evidence that wildfire smoke exposure is associated with all-cause mortality, although there has not been a consistent association between smoke exposure and respiratory mortality or any other specific cause for mortality [71]. Newer data from a large case-crossover study in Washington show an overall 1.3% increase in (non-traumatic) mortality on the day of and day after wildfire smoke exposure and an increase in COPD-specific mortality of 14% after the exposure [58].

In addition to impacting air quality, wildfires also emit pollutants, including human carcinogens, that can contaminate water and soil and thus remain in the environment long after PM_2.5_ returns to baseline levels [82]. There is an established link between total ambient air pollution and lung cancer. In 2013, the International Agency for Research on Cancer (IARC) classified air pollution as a group 1 human carcinogen causing lung cancer [54]. There is a 9% increase in lung cancer incidence or mortality for every 10 µ/m^3^ increase in PM_2.5_ [54], and among never smokers, ambient air pollution exposure is associated with lung cancer with OR 1.79 [87]. The association with lung cancer also appears to be specific to wildfire smoke exposure. The risk of lung cancer in firefighters exposed to wildfire smoke has been estimated at 8 to 43% [88], and in 2022, the IARC classified wildfire occupational exposure as carcinogenic to humans [89]. In an observational cohort study in Canada where individuals were followed from 1996 to 2015, exposure to wildfires within a 50 km radius was associated with a 4.9% higher incidence of lung cancer [82]. 

Total ambient PM_2.5_ is causally associated with cardiovascular disease [90]; however, data linking wildfire smoke PM_2.5_ and cardiovascular outcomes are conflicting [31]. While numerous studies examining cardiac outcomes with wildfire PM exposure were negative [71], Magzamen et al. showed an increase of 10 µg/m^3^ in wildfire smoke PM_2.5_ is associated with cardiovascular (cerebrovascular, heart failure, ischemic heart disease) hospitalization 2–3 days after initial exposure, and cardiac arrest deaths on the day of first fire exposure (lag day 0) [64]. Other data demonstrate increased healthcare utilization for congestive heart failure and ischemic heart disease after wildfire PM_2.5_ exposure [91,92]. Thilakaratne et al. show a 0.89% increased risk (95% CI 0.8–0.98%) in cardiovascular ED visits with wildfire PM_2.5_ exposure [75]. Underlying diabetes may increase cardiorespiratory morbidity from smoke exposure [72]. However, the overall data linking wildfire smoke and cardiovascular outcomes remains too limited and inconsistent to make definitive conclusions, and further research is needed [71]. 

Wildfire smoke exposure has been linked to infectious diseases. Increased bronchitis and acute respiratory infection after wildfire smoke exposure have been described [72]. Fungal infections such as coccidioidomycosis may spike after wildfire [44,79]. In a case-crossover study, wildfire-associated PM_2.5_ exposure was associated with 23% higher odds of active tuberculosis diagnosis, which the authors propose is due to increased risk of TB reactivation since exposure to inhaled pollutants such as tobacco smoking is an established risk factor for TB reactivation [80].

The acute symptoms related to smoke exposure affect not only healthcare utilization and quality of life but also importantly impact work productivity. In a large survey study in California, exposure to wildfire smoke was associated with short-term work loss due to sickness. The odds ratio of work loss was 1.45 when the PM_2.5_ exposure exceeded 12 µg/m^3^ [93]. Wildfire smoke exposure can also have a detrimental on mental health, including anxiety, depression, social isolation, and post-traumatic stress [94]. 

## 6. Vulnerable Populations

Wildfire smoke has worse effects on several vulnerable populations, including children, older individuals, women, socioeconomically disadvantaged, and occupationally-exposed groups. Children are more susceptible to smoke exposure due to greater minute ventilation per unit body weight, smaller airways, more time outdoors, and developing lungs [95,96]. Little is known about the long-term effects of wildfire exposures in utero or very early in life; however, data suggests that prenatal exposure to wildfire PM_2.5_ is associated with higher rates of adverse obstetrical outcomes such as low birth weight and preterm birth [79,97,98]. Although not conclusively proven, it is plausible that PM_2.5_ exposure during critical developmental periods could impair lung growth [96]. A retrospective study demonstrated that smoke exposure in the first trimester of pregnancy and in the postnatal period was associated with earlier pediatric treatment for the first upper respiratory infection [99]. 

Several studies examining the impact of wildfire smoke on asthma-related hospitalization find the population most affected is aged 0 to 5 [25,59,70]. In a retrospective study of over fifty thousand children with asthma in Calgary, Canada, wildfire smoke days were associated with a relative risk for asthma exacerbation of 1.13 over baseline, a risk that was not seen with general ambient air pollution exposure [100]. Henry et al. demonstrate an increase in asthma emergency department visits and hospitalization in children, especially those under 5 years old, within the first three days of wildfire smoke exposure [70]. In a separate pediatric cohort, there was a higher incidence not only of asthma exacerbations but also bronchitis and acute respiratory infection after incremental increases in wildfire smoke exposure [72]. Li et al. confirmed these findings in a case-crossover study of over thirty-six thousand children under the age of five and demonstrated a 1 µg/m^3^ increase in wildfire-sourced PM_2.5_ led to a 3.2% increased risk of acute respiratory infection—an effect equivalent to that of 5 µg/m^3^ of non-fire PM_2.5_ [81]. A questionnaire study of children with and without asthma exposed to wildfire smoke in California showed an association between smoke exposure days and symptoms (nose, eye, and throat irritation; cough; wheezing), medication use, and physician visits. The associations were more pronounced among children without asthma, possibly because this group generally takes fewer precautions against exposure as compared to children with asthma [101]. 

Older adults are more susceptible to the adverse effects of smoke due to a higher prevalence of pre-existing cardiovascular and respiratory conditions and reduced clearance of PM_2.5_ from the respiratory tract with age [25]. Wildfire smoke exposure is associated with an 11% increase in physician visits in an older population [72]. A study within the Medicare population showed that PM_2.5_ from smoke days was associated with asthma exacerbation and wheezing, and while both smoke and non-smoke PM_2.5_ exposure was associated with increased hospitalization, asthma hospitalization was higher on smoke days [65]. In a model of wildfire exposures in California from 2007 to 2018, 56% (or 2.7 million) of California residents over 65 years old lived in counties representing the top three quartiles of wildfire PM_2.5_ emissions [102]. Older adults are thus highly exposed, more susceptible to health effects, and may also be limited in their ability to evacuate wildfire-affected areas [103].

Several studies suggest a sex-specific difference in asthma exacerbations and healthcare utilization following wildfire smoke PM_2.5_ exposure. Reid et al. show the effect of wildfire PM_2.5_ on increased asthma ED visits and hospitalization was most evident in women aged 20–64 [13]. Similarly, in a study demonstrating an overall 1.96% increase in asthma ED visits after wildfire PM_2.5_ exposure, the effect was more pronounced in women aged 20 and older, who had a 5.08% increase in asthma ED visits [66]. Delfino et al. also showed a significant increase in asthma admissions during and after wildfire exposure in women ages 5–64 but not in men [104]. A meta-analysis of within-study relative risks highlighted a greater impact of wildfire smoke on women as compared to men with asthma (OR 1.035) and COPD (OR 1.018) [105]. In an elderly population, wildfire smoke exposure was associated with more respiratory-related hospitalizations in women (10.4%) than in men (3.7%) [106]. As outlined by Reid et al., it remains unclear whether women with asthma have greater biologic susceptibility to wildfire smoke, are more likely to have baseline uncontrolled asthma, or are more likely to seek medical care for asthma-related symptoms than men [13]. 

Disparities in socioeconomic status can play a role in varying exposures to wildfire smoke among different communities. Smoke exposure has increased overall in the United States from 2011–2021; however, disadvantaged communities experienced the largest increases in exposure [107]. In addition, people of color bear a disproportionate burden of asthma [108] and are thus potentially more susceptible to health effects from exposure to wildfire smoke. In a study comparing estimates of wildfire PM_2.5_ exposure and hospital admissions in a population over the age of 65, Liu et al. found significantly more respiratory hospital admissions for Blacks (21.7%) than whites (6.9%) [106]. 

Firefighters are a unique population who are subject to intense wildfire smoke exposure, and the effects of smoke exposure can be exacerbated by the increased minute ventilation during work and the limited practicability of using personal protective respirators during such physical activity [3,109]. Respiratory risks to firefighters include impaired lung function, asthma, and lung cancer; firefighters are also at higher risk for hypertension, cardiovascular disease, and post-traumatic stress [74,88,110]. Outdoor workers who are not firefighters also have substantial exposure to smoke, and the state of California has taken measures to protect these workers by requiring employers to monitor and communicate the air quality index (AQI) when workers may be exposed to wildfire smoke and to provide respirators when the air quality is hazardous [109]. 

## 7. Strategies for Risk Reduction and Mitigation

Numerous interventions for wildfire smoke risk reduction are suggested by public health organizations, including limiting outdoor exposure during poor air quality days, using air quality alert systems to plan activities, wearing a facemask, and using air filtration systems [111,112,113]. During wildfires, indoor PM_2.5_ levels are approximately 20% of the measured outdoor levels [114]; thus, it is generally recommended that people monitor air quality and avoid vigorous outdoor activity in favor of spending time indoors in air-conditioned buildings. Public health organizations recommend wearing a face mask to reduce wildfire smoke exposure, and although facemasks do not confer protection from toxic gases in smoke, such as carbon monoxide and VOCs, a modeling study shows that the use of N95 respirators could reduce the risk of COPD exacerbations and hospitalizations during air pollution events such as wildfires [115,116]. At the building level, closing windows and doors along with the use of air filtration with high-efficiency particulate air (HEPA) filters can reduce exposure. However, even indoors, infiltration of PM_2.5_ can occur during a smoke episode, as demonstrated by a study using indoor sensors at a healthcare facility, highlighting the need to monitor and reduce indoor exposure around those at the highest risk for adverse effects from smoke [117]. Air filtration devices can reduce indoor PM_2.5_ concentrations by 50%, and in a randomized controlled trial comparing air filtration to a sham device, filtration was associated with a 70% reduction in risk of COPD exacerbation, with less rescue inhaler use and improved symptoms. At the community level, risk mitigation interventions include actions such as school closures, establishment of public clean-air shelters, implementation of stay-at-home orders, and issuance of evacuation orders [3]. 

There are barriers to implementing recommendations to reduce wildfire smoke exposure. Many of the self-protective measures are costly, potentially restricting access to people based on resources [107]. Individual-level interventions, including the use of N95 respirators and avoidance of outdoor activities, may not be adopted due to misunderstanding or not receiving communication [118]. Data show that people often do not recall the technical recommendations of a fire-related public health message and do not follow the suggested recommendations; however, promoting communication with peers helps to engender behavioral change [119]. It is also critical to recognize members of some communities, for example rural or tribal, may be hesitant to trust information from state and federal agencies [118]. The most effective messages use simple language that is easily recalled and bidirectional communication with stakeholder engagement, e.g., in town hall meetings. Study shows the communication needs to be context-specific, for example, recognizing some populations, such as the elderly, may not respond to social media outreach [120]. In a survey of patients with asthma and COPD, the most common wildfire smoke mitigation strategy used was closing windows and staying indoors. Most individuals had not received any advice from their medical provider about the use of masks and did not use a mask [115]. An analysis of behavioral change during wildfire events, using internet searches and smartphone-derived location data movement, showed those in higher socioeconomic groups more readily followed stay-at-home recommendations, were more likely to seek information about protective measures, and were more likely to have indoor pollution monitors. However, data from these indoor particulate matter monitors (Purpleair) showed people were still exposed to very high levels of indoor smoke during smoke events [121]. 

The integration of wildfire prediction systems could play a future role in calculating exposure and help guide public health response. For instance, predictions of increased PM_2.5_ from the “BlueSky” wildfire forecasting system in British Columbia, Canada, correlated with an uptick in bronchodilator prescriptions and asthma-related physician visits [122]. Future study is needed to determine if public health guidance based on predictive smoke models brings about behavioral change and improved health outcomes.

Reliance on individual protection from wildfire smoke is likely biased against disadvantaged groups and is in contrast to the public health approach to ambient pollution exposure, where efforts to reduce pollutants from the source have diminished inequalities in exposure [121]. Therefore, novel approaches to fire management are needed. Fire suppression alone delays the health effects but ultimately results in more people affected and greater intensity of exposure [123]. Managed burning, controlled burning, and thinning can limit flammable fuels. Data suggest that more frequent low-intensity fires and prescribed burns result in significantly lower relative exposure. Practice is changing: prescribed fires have increased 100-fold from 1998 to 2018 [89]. Utilizing prescribed fires to reduce the risk of uncontrolled fires in areas with large fuel accumulation could be beneficial, but systematic investigation is needed to determine if this strategy results in less smoke exposure and fewer adverse health effects [3]. 

## 8. Future Directions

Our review highlights the evolving global problem of wildfires, and the growing data showing adverse respiratory health effects from wildfire smoke exposure. To tackle this mounting public health crisis, multiple working groups have emphasized the importance of fostering cross-discipline communication and collaboration between the forestry, fire, and health fields [3,4]. There is an unmet need to develop evidence-based risk mitigation strategies and to determine the best processes for public health communication in the community. Future research should also include determining the best means for assessing wildfire toxic exposures, which may include improved satellite imaging, atmospheric modeling, and better emissions data collection. The risk/benefit of controlled burns warrants scientific evaluation. Additional topics of interest for further study include analysis of wildfire-specific toxins and means of lung injury and carcinogenesis, exploration into the signal of sex-specific outcomes, and long-term investigation into lung function outcomes and respiratory mortality after acute or repeated wildfire smoke exposure.

## 9. Conclusions

Wildfires are increasing in frequency and severity, and due to climate change and human activities are predicted to become an ever-present phenomenon with widespread impact on health. Wildfire PM_2.5_ injures the lungs and begins a cascade of inflammatory processes that lead to exacerbation of obstructive lung disease and small airways remodeling. Wildfires have acute adverse effects on people living with asthma and COPD, including exacerbations, emergency department visits and hospitalizations, and increased burden of respiratory symptoms. Wildfires may have long-term effects on lung function, risk for lung cancer, and mortality. Risk mitigation needs to be approached from an individual level, population level, and planetary health level.

## Figures and Tables

**Table 1:** Epidemiologic evidence for adverse respiratory health effects associated with wildfire smoke exposure.

Adverse Respiratory Health Effects	Reference Number
Asthma hospitalization	[13,59,61,62,63,64,65]
Asthma emergency department visit	[13,25,26,59,61,62,66,67,68,69,70]
Asthma symptoms	[71,72,73]
Asthma incidence	[74]
COPD hospitalization	[13,61,63,64]
COPD emergency department visit	[13,26,75]
COPD symptoms	[76]
Long-term impact on lung function	[8,77,78]
Respiratory infection	[44,72,79,80,81]
Lung cancer incidence	[82]
Respiratory mortality	[58]

## Data Availability

Not applicable, no new data were created.
